# The Function of miRNAs in Plants

**DOI:** 10.3390/plants9020198

**Published:** 2020-02-05

**Authors:** Anthony A Millar

**Affiliations:** Division of Plant Science, Research School of Biology, The Australian National University, Canberra ACT 2601, Australia; tony.millar@anu.edu.au

**Keywords:** miRNAs, development, abiotic stress, nutrient availability, circular RNAs, tasiRNA, phasiRNA

## Abstract

MicroRNAs (miRNAs) are a class of small RNAs (sRNAs) that repress gene expression via high complementary binding sites in target mRNAs (messenger RNAs). Many miRNAs are ancient, and their intricate integration into gene expression programs have been fundamental for plant life, controlling developmental programs and executing responses to biotic/abiotic cues. Additionally, there are many less conserved miRNAs in each plant species, raising the possibility that the functional impact of miRNAs extends into virtually every aspect of plant biology. This Special Issue of *Plants* presents papers that investigate the function and mechanism of miRNAs in controlling development and abiotic stress response. This includes how miRNAs adapt plants to nutrient availability, and the silencing machinery that is responsible for this. Several papers profile changes in miRNA abundances during stress, and another study raises the possibility of circular RNAs acting as endogenous decoys to sequester and inhibit plant miRNA function. These papers act as foundational studies for the more difficult task ahead of determining the functional significance of these changes to miRNA abundances, or the presence of these circular RNAs. Finally, how miRNAs trigger the production of secondary sRNAs is reviewed, along with the potential agricultural impact of miRNAs and these secondary sRNA in the exemplar crop maize.

## 1. Introduction

MicroRNAs (miRNAs) have now been linked to most aspects of plant biology. They were first identified in plants less than 20 years ago, but they have been shown to be critical regulators of developmental process such as leaf morphogenesis, vegetative phase change, flowering time and response to environmental cues. This Special Issue presents a collection of papers that continues the molecular and functional characterization of plant miRNAs, as well as reviews that reflect on past achievements and outline the challenges and opportunities that lie ahead.

## 2. Development

Plant miRNAs are probably best known for their role in development. Many of the ancient miRNAs regulate highly conserved transcription factors or other regulatory genes that are fundamental in the development of terrestrial plants. MiR171 is one of these ancient miRNAs, being present in all lineages of land plants, where they negatively regulate genes encoding GRAS-domain SCARECROW-like transcription factors, but the functional outcome of this regulation is yet to be determined in many plant species. Part of the reason is that most plant miRNAs correspond to families of multiple redundant genes, and this is the case for miR171 in tomato, which has 11 family members. Kravchik et al. [1] investigate miR171 function in tomato using short tandem target mimic (STTM) technology, expressing a decoy that binds and sequesters all miR171 isoforms to inhibit the entire family [2]. They show miR171 in tomato is involved not only in shoot branching and leaf morphogenesis, but also in male development, as *STTM171* tomato plants had altered tapetal development and, consequently, altered pollen ontogenesis. Consistent with other species, miR171 appears to have diverse roles in tomato development.

## 3. Environmental Response

### 3.1. Abiotic Stress

To gain insights into whether an miRNA is involved in a stress response, the most obvious experiment is to determine whether its abundance changes under a certain stress. First, Pegler et al. [3] performed RNA-seq to determine the abundance of miRNAs in *Arabidopsis* under heat, drought, and salt stress conditions. This global survey identified many miRNAs with high-level fold changes under these conditions, thus identifying miRNAs that are candidates for playing important functional roles during these stresses. That study will act as a fundamental resource for future studies. Drought stress and water use efficiency will be a future key crop trait. Njaci et al. [4] identified miRNAs that alter in abundance under extreme water deficit in *Tripogon loliiformis*, a plant that can resurrect from a desiccated state. They found many conserved miRNAs differentially accumulated in roots and shoots during dehydration, likely reflecting the broad changes to metabolism and physiology during this extreme stress. Again, this study will act as a foundation for the investigation of miRNAs in desiccation tolerance, with the ultimate goal to utilize these for introducing tolerance into important crop species. In a more targeted study, López-Galiano et al. [5] focused on examining the change in miR159 abundance in tomato during drought, where they found miR159 to be downregulated during stress, while the mRNA levels of its corresponding target gene were derepressed. All these studies represent the start of the investigation of how miRNAs respond to stress and how they could be possibly utilized for developing stress tolerance. The much more challenging process lies ahead of determining what is the functional impact of these miRNA abundance changes and whether this information can be used to engineer stress tolerance into crop species. Indeed, despite miR159 being one of the earliest identified and most extensively studied miRNA, no clear conserved functional role for this miRNA has been identified. Millar et al. [6] summarize the literature concerning the biology of miR159, discussing the various potential functions that have been identified, and the questions that need to be addressed concerning this miRNA.

### 3.2. Nutrient Availability

MiRNA abundances also respond to nutrient availability. One such nutrient is copper (Cu), and four different miRNAs are known to respond to Cu levels, namely, miR397, miR398, miR408, and miR857, all highly conserved plant miRNAs. In this Special Issue, Shahbaz and Pilon [7] present an STTM which inhibited miR397, miR398, and miR408 simultaneously, resulting in higher levels of their target mRNAs under Cu-limiting conditions. The targets are all Cu-containing proteins, and failure to repress these targets under Cu-limiting conditions indirectly leads to reduced levels of an unrelated Cu-containing protein, plastocyanin, in the STTM transgenic plant lines. As plastocyanin is key for photosynthesis, this likely explains a decrease in photosynthetic electron transport activity of the STTM lines under Cu-limiting conditions, leading to a decrease in plant biomass. Therefore, the authors have superbly shown how these miRNAs regulate the Cu economy, channeling Cu to the most important Cu-containing proteins during Cu limitation.

One of the most limiting nutrients worldwide is phosphorous (P). Peglar et al. [8], investigate the machinery that is needed for the miR399-mediated regulation of *PHOSPHATE2* (*PHO2*). They show that in *Arabidopsis*, of the four members of DOUBLE-STRANDED RNA BINDING (DRB) protein family, DRB1 is the main player involved in the miR399 regulation of *PHO2*, but that DRB2 and DRB4 also play minor roles, and this regulation involves both an mRNA cleavage and translational repression mechanism. All these mechanisms are required to maintain *Arabidopsis* P homeostasis and highlight the complexity of this process.

## 4. Complexity of miRNA Regulation

MiRNA function can itself be regulated by RNAs where, in plants, noncoding RNA transcripts containing miRNA binding sites have been shown to act as decoys or miRNA target *MIMIC*s, to sequester and inhibit miRNA function [9]. In animals, such RNAs are called competitive endogenous RNAs (ceRNAs), and some of the first identified were circular in form and contained multiple miRNA binding sites. It was thought that being circular increased stability and the effectiveness of being a decoy, but whether such RNAs exist in plants is unknown. In this Special Issue, Capelari et al. [10] bioinformatically mined publicly available RNA-seq data from ARGONAUTE-immunoprecipitation libraries (AGO-IP) and identified 1000s of potential circular RNAs, many of which contain potential miRNA binding sites. As many of the corresponding target mRNAs were found to be enriched in these AGO-IP libraries, this suggested that circular ceRNAs could be operating in plants. Obviously, more work is needed in confirming this, but in this intriguing paper, the authors have identified strong candidates to pursue.

In addition, miRNAs have been found not only to silence target transcripts through slicing, but in some instances slicing triggers the production of secondary siRNAs, known as trans-acting siRNAs (tasiRNA) or phased siRNAs (phasiRNAs). De Felippes [11] reviews the complex mechanisms and hypotheses by which tasiRNAs and phasiRNAs are generated, the factors involved, the regulatory advantages of transitivity, and the potential use of the natural amplification process to result in strong artificial gene silencing.

## 5. Application of miRNAs to Agriculture

Given miRNAs’ central role in plant development via target key regulatory genes, and their potential role in stress response, their manipulation has the potential to alter key agronomic traits. Zhang et al. [12] summarizes the different gene silencing pathways and core machinery in maize, and their function in maize biology, detailing the traits that miRNAs, phasiRNAs, and tasiRNAs regulate and their potential use in agronomic improvement of maize, be it developmental timing, plant architecture, sex determination, fertility, or abiotic stress resistance. This gives an overview to the many potential applications in just one plant species. Given our extensive knowledge on the fundamental biology of plant miRNAs in model species, the future trajectory of this field will be their application in important crop species, where understanding their role and applying this knowledge have real potential for important agronomic outcomes.
